
JOURNAL INFORMATION
==============================
NLM Title Abbreviation: Sci China Life Sci
Journal ID: Sci China Life Sci
NLM Journal ID: 101529880

Science China. Life Sciences

ISSN: 1674-7305
EISSN: 1869-1889

ARTICLE INFORMATION
==============================
PMCID: PMC7089321
PMID: 25558865
DOI: 10.1007/s11427-014-4794-z
Article ID: 4794
Article version: 1
Subjects: Letter to the Editor

May early intervention with intravenous immunoglobulin pose a potentially successful treatment for Ebola virus infection?

Ge Ying
Li TaiSheng litsh@263.net

grid.410318.f 0000000406323409 Department of Infectious Diseases, Peking Union Medical College Hospital, Chinese Academy of Medical Science, Beijing, 100730 China
Electronic publication date: 2014 Dec 22
Print publication date: 2015
Volume: 58
Issue: 1
First page: 108
Last page: 110
Received 2014 Nov 7; Accepted 2014 Nov 22
Copyright: © The Author(s) 2014
License: This article is published under license to BioMed Central Ltd. Open Access This article is distributed under the terms of the Creative Commons Attribution License which permits any use, distribution, and reproduction in any medium, provided the original author(s) and source are credited.
License URL: https://creativecommons.org/licenses/by/4.0/
License: Open AccessThis article is distributed under the terms of the Creative Commons Attribution 2.0 International License (https://creativecommons.org/licenses/by/2.0), which permits unrestricted use, distribution, and reproduction in any medium, provided the original work is properly cited.
License URL: https://creativecommons.org/licenses/by/2.0/

Keywords: Severe Acute Respiratory Syndrome, Capillary Leak Syndrome, Central Nervous System Complication, Severe Acute Respiratory Syndrome Coronavirus, Convalescent Plasma
issue-copyright-statement: © Science in China Press and Springer-Verlag GmbH 2015

==============================

Dear Editor,

Ebola virus is negative-sense, single stranded RNA virus of the family Filoviridae. Since their discovery in 1976, Ebola viruses have caused numerous outbreaks of severe hemorrhagic fevers in Africa. The emergence of Ebola virus disease (EVD) in Guinea and its spread to urban centers in neighboring West African countries recently has caused international alarm. These filovirus infections are characterized by acute onset of illness after an incubation period of 2–21 days, with initial symptoms of fever, chills, myalgia, and malaise. The disease signs and symptoms frequently become more severe; many patients have features of systemic inflammatory response syndrome, such as fever, tachycardia and hyperventilation with less frequent leukopenia. Organ dysfunction (severe sepsis) and shock are also common; however, the capillary leak syndrome and subsequent secondary lung injury and hypoxia are much less apparent than with typical bacterial sepsis including. The terminal phase results in death or a prolonged convalescent period, depending on various host and viral factors. Death by shock and multi-organ failure typically occurs within 6–10 days after symptom onset. The case-fatality rate of EVD has been 50%–60% [1].

Currently, no specific therapy is available that has demonstrated efficacy in the treatment of Ebola hemorrhagic fever. The search for a filovirus therapeutic has been historically challenging. Conventional antiviral strategies, such as ribavirin, recombinant IFN-α, and treatment with convalescent antisera have proven ineffective in animal trials; however, there are examples where different approaches have demonstrated a survival benefit. Nucleoside analogue inhibitors of the cell-encoded enzyme S-adenosylhomocysteine hydrolase (SAH) have been shown to inhibit Zaire ebolavirus replication in adult BALB/c mice infected with mouse-adapted Ebola virus. Inhibition of SAH indirectly inhibits transmethylation reactions required for viral replication. Smith et al. [2] found that in rhesus macaques infected with a lethal dose of Ebola virus, treatment with interferon beta early after exposure led to a significant increase in survival time, though it did not reduce mortality significantly. Passive immunity has been attempted by using equine-derived hyperimmune globulins and human-derived convalescent immune globulin preparations. In Ebola virus-infected cynomolgus macaques, use of human recombinant interferon α-2b in conjunction with hyperimmune equine immunoglobulin G (IgG) delayed but did not prevent death [3]. During the 1995 outbreak in Kikwit, Democratic Republic of Congo, human convalescent plasma was used to treat eight patients with proven Ebola disease, and only one patient died. Subsequent studies could not demonstrate survival benefit conferred by convalescent plasma products. The survival of these patients suggests that passive immunity may be of benefit in some patients. DNA vaccines expressing either envelope GP or nucleocapsid protein (NP) genes of Ebola virus have been demonstrated to induce protection in adult mice exposed to the virus. These vaccines were administered by coating gold beads with DNA expressing the genes for either GP or NP, and they were delivered by skin particle bombardment using a PowderJect-XR gene gun. Both vaccines induced measurable antibody responses detected by enzyme-linked immunosorbent assay and induced cytotoxic T-cell immunity [4]. Other experimental therapies that use available drugs, though not approved by the US Food and Drug Administration for treatment of Ebola virus infection, may be considered. Agents that may reduce mortality without directly effecting viral replication include activated protein C and a recombinant nematode anticoagulant protein (NAP) that inhibits activated factor VII-tissue factor complex. NAP resulted in attenuation of the coagulopathy associated with decreased fibrinolysis and fibrin deposition with a resultant decrease in the severity of the systemic inflammatory response syndrome. All of the strategies described above that provided protection were administered either before or shortly after a lethal challenge. In the absence of any licensed prophylaxis or treatment, supportive care for individual patients has been fraught with difficulty and evoked widespread suspicion and fear in affected communities. Current procedures for dealing with suspected or confirmed cases include disrupting virus transmission in the form of quarantine and barrier protection, and palliative care. As yet, there has been no sign the outbreak is abating.

The pathogenesis of EVD in human remains poorly understood. The usual study model is infection of non-human primates, while other valuable insights gained from rodent models are constrained by differences in clinical features. A common pathogeznic feature of viral haemorrhagic fever viruses is their ability to disable the host immune response by attacking and manipulating the cells that initiate the antiviral response [5]. End organ dysfunction in EVD results from a combination of direct injury of virus-infected tissues and the indirect effects of the immune response. Within an outbreak, viral strain variation is limited and host response is the main determinant of outcome. Recovery from Ebola infection is associated with early and well-regulated inflammatory responses, including early antibody production, reduced T cell apoptosis and more rapid clearance of viraemia compared to fatal cases. Strategies that provide cheap, easily administered, and potent virus inhibition in a patient displaying EVD remain elusive. We may get some clues from previous observations. A large outbreak of human enterovirus 71 (EV71)-associated hand-foot-and-mouth disease (HFMD) in China from March through May 2008 resulted in 61,459 cases, including 38 deaths. The clinical characteristics of this outbreak in China were mainly associated with central nervous system (CNS) involvement. We systematically compared the changes in peripheral lymphocyte subsets in EV71-infected patients with and without CNS complications. The results demonstrated a significant increase in the absolute count and percentage of B lymphocytes in children with symptoms of encephalitis at the acute stage, followed by a rapid recovery of the parameters to normal levels 2–3 weeks later during the convalescent stage. IgG levels were significantly higher in the CNS group compared to the mild cases [6]. It appears that the immunophenotypic changes are associated with the severe clinical manifestations of HFMD. We hypothesize that patients with severe EV71 infections rapid recovery of CNS complications were related to the restoration of IgG levels or B cells during the acute phase under Intravenous immunoglobulin (IVIg) treatment. In previous studies, IVIg therapy shown to be susceptible to chronic enterovirus infections and administration of IVIg could ameliorate disease. Immunoglobulins are large Y-shaped proteins produced by B-cells and plasma cells. IVIg is used at replacement dose (200–400 mg $kg^{-1}$ 3 weekly) to treat patients with primary antibody deficiencies, while high-dose IVIg is used as an immunomodulatory agent in a wide range of autoimmune and inflammatory disorders as well as bacterial and viral infections that do not respond to conventional therapy. IVIg is prepared from pooled plasma from a high number of donors which are frequently used off label in many autoimmune conditions. Several mechanisms of action of IVIg have been proposed, including maturation of antigen-presenting cells, induction of regulatory T-cell subsets and a general anti-inflamatory capacity. More recent data suggest that the presence of "natural antibodies" may be key to biological relevant IVIg activities by enhancing the maintenance of cellular and tissues homeostasis via stimulation of cell surface receptors [7]. Gorochov et al. [7] identified large plasma-cell mobilization exclusively in IVIg-treated autoimmune patients 7 days after initiation of treatment. The majority of IVIG-mobilized plasma cells were immature HLA-DRhigh/CD138low/CXCR4low plasma cells expressing intracellular immunoglobulin G which were neither IVIg- nor human IgG-specific. Strong negative correlation noted between the absolute number of IVIg-mobilized plasma cell and time to improve neurological function of Guillain-Barre syndrome (GBS). They made a conclusion that IVIg promotes immature plasma cell mobilization in patients with GBS, and prominent day 7 plasma- cell mobilization is a favorable prognostic marker in patients with GBS receiving IVIg treatment.

We have to seek optimal antiviral strategies which could easily obtain and could afford in underdeveloped countries. The use of IVIg as a replacement therapy in patients with primary Ab deficiency is still the main indication in the field of infectious disease treatment and prevention. It is likely that the modulation of the inflammatory response in tissues affected by viral pathogens also plays an important role in modulating the disease. Hyperimmune immunoglobulin has been used successfully to treat WNV-induces encephalitis [8]. IVIg reduces mortality in combination with antiviral drugs in adult HSCT recipients with respiratory syncytial virus infection if the IVIg therapy was initiated before the onset of severe respiratory failure [9]. Antipathegen-specific and immune-modulatory effects may supplement each other. IVIg may help to re-equilibrate the relative proportions of specific and naturally occurring Abs (NoAbs), and this could be the base of beneficial effects observed in patients with HIV or H1N1 influenza infection [10]. Therefore, we can also make a hypothesis that EVD could also benefit with the intervention of IVIg in the early stage of the disease. No experimental results could learn from EVD treatment in the past several decades. We should not just wait for it to vanish spontaneously.

IVIg is currently used in a wide range of clinical conditions. It may probably also work in viral infections, especially the virus which does not respond to conventional antiviral strategies, such as Ebola virus. The clinical relevance concerning the mechanisms of action has been highlighted although the knowledge of molecular mechanisms in specific conditions is relatively limited.

**Open Access** This article is distributed under the terms of the Creative Commons Attribution License which permits any use, distribution, and reproduction in any medium, provided the original author(s) and source are credited.

This article is published with open access at link.springer.com


References

1 Towner JS Sealy TK Khristova ML Albariño CG Conlan S Reeder SA Quan PL Lipkin WI Downing R Tappero JW Okware S Lutwama J Bakamutumaho B Kayiwa J Comer JA Rollin PE Ksiazek TG Nichol ST Newly discovered Ebola virus associated with hemorrhagic fever outbreak in Uganda PLoS Pathog 2008 4 e100212 10.1371/journal.ppat.1000212 PMC2581435 19023410
2 Smith LM Hensley LE Geisbert TW Johnson J Stossel A Honko A Yen JY Geisbert J Paragas J Fritz E Olinger G Young HA Rubins KH Karp CL Interferon-ß therapy prolongs survival in rhesus macaque models of Ebola and Marburg hemorrhagic fever J Infect Dis 2013 208 310 318 10.1093/infdis/jis921 23255566 PMC3685222
3 Sullivan NJ Sanchez A Rollin PE Yang ZY Nabel GJ Development of a preventive vaccine for Ebola virus infection in primates Nature 2000 408 605 609 10.1038/35046108 11117750
4 Elshabrawy HA Fan J Haddad CS Ratia K Broder CC Caffrey M Prabhakar BS Identification of a broad-spectrum antiviral small molecule against severe acute respiratory syndrome coronavirus and Ebola, Hendra, and Nipah viruses by using a novel high-throughput screening assay J Virol 2014 88 4353e65 10.1128/JVI.03050-13 24501399 PMC3993759
5 Mahanty S Bray M Pathogenesis of filoviral haemorrhagic fevers Lancet Infect Dis 2004 4 487 498 10.1016/S1473-3099(04)01103-X 15288821
6 Xie J Jiao Y Qiu ZF Li Q Li TS Significant elevation of B cells at acute stage in enterovirus 71-infected children with central nervous system involvement Scand J Infect Dis 2010 42 931 935 10.3109/00365548.2010.498018 20950217
7 Mori I Parizot C Dorgham K Demeret S Amoura Z Bolgert F Gorochov G Prominent plasmacytosis following intravenous immunoglobulin correlates with clinical improvement in Guillain-barre syndrome PLoS One 2008 3 e2109 2118 10.1371/journal.pone.0002109 18461177 PMC2362102
8 Sundblad A Marcos MA Malanchere E Castro A Haury M Huetz F Nobrega A Freitas A Coutinho A Observations on the mode of action of normal immunoglobulin at high doses Immunol Rev 1994 139 125 158 10.1111/j.1600-065X.1994.tb00860.x 7927409
9 Makhoul B Braun E Herskovitz M Ramadan R Hadad S Norberto K Hyperimmune gammaglobulin for the treatment of West Nile virus encephalitis Isr Med Assoc J 2009 11 151 153 19544704
10 Shen X Tomaras GD Alterations of the B-cell response by HIV-1 replication Curr HIV/aids Rep 2011 8 23 30 10.1007/s11904-010-0064-2 21161615 PMC3638746
